# The Coronavirus Pandemic and Lessons Learned in Preschools in Norway, Sweden and the United States: OMEP Policy Forum

**DOI:** 10.1007/s13158-020-00267-3

**Published:** 2020-06-17

**Authors:** Ingrid Pramling Samuelsson, Judith T. Wagner, Elin Eriksen Ødegaard

**Affiliations:** 1 Department of Education, Communication and Learning, Gothenburg University, Box 300, 405 30 Gothenburg, Sweden; 2 Emerita Professor of Education and Child Development, Broadoaks Children’s School, Whittier, CA USA; 3 KINDknow Research Center, Western Norway University of Applied Sciences, Bergen, Norway

**Keywords:** Preschool, Pandemic, Novel coronavirus, COVID-19

## Abstract

The novel coronavirus, also known as COVID-19, has moved rapidly across the world in 2020. This article reports on the recent consequences of the pandemic for early childhood education in Sweden, Norway, and the United States. The authors illustrate the effects of the pandemic on preschools in their countries, against a backdrop of frequent changes in infection and mortality rates, epidemiological understandings, government strategies, and mitigation strategies regarding preschool closures. Teachers report their experiences and actions in specific early childhood education settings, across the three national contexts. These experiential snapshots identify program priorities, parents’ and children’s reactions, and the commitment and concerns of teachers. The conversations reveal culturally situated similarities of early childhood educational practices but also differences across contexts. Teachers report on the challenges of their experiences but also benefits for their practice and how they engage with children and their families. Ideas about future preparedness for such pandemics are also discussed.

## Résumé

Le nouveau coronavirus, également connu sous le nom de COVID-19, s’est déplacé rapidement à travers le monde en 2020. Cet article rend compte des conséquences récentes de la pandémie pour l’éducation de la petite enfance en Suède, en Norvège et aux États-Unis. Les auteurs analysent les effets de la pandémie sur les établissements préscolaires dans leurs pays, dans un contexte de changements fréquents des taux d’infection et de mortalité, de compréhension épidémiologique, de stratégies gouvernementales et de stratégies d’atténuation au regard des fermetures d’établissements préscolaires. Les enseignants font part de leurs expériences et de leurs actions dans des milieux spécifiques d’éducation de la petite enfance, dans les trois contextes nationaux. Ces instantanés expérientiels identifient les priorités du programme, les réactions des parents et des enfants, ainsi que l’engagement et les préoccupations des enseignants. Les conversations révèlent des similitudes culturelles des pratiques éducatives en éducation de la petite enfance, mais aussi des différences selon les contextes. Les enseignants rendent compte de défis de leurs expériences, mais aussi de bénéfices pour leur pratique et de la façon dont ils interagissent avec les enfants et leurs familles. Des idées sur la préparation future à de telles pandémies sont également discutées.

## Resumen

El nuevo virus corona, conocido también como COVID-19, se ha movido rápidamente por todo el mundo en el 2020. Este artículo informa sobre las consecuencias de la pandemia sobre la educación temprana en Suecia, Noruega, y los Estados Unidos. Los autores muestran los efectos de la pandemia en los establecimientos preescolares en sus países en un contexto de cambios frecuentes de las tasas de infección y mortalidad, entendimientos epidemiológicos, estrategias gubernamentales, y estrategias de mitigación relacionadas con el cierre de los recintos preescolares. Los maestros reportan sus experiencias y acciones en ambientes específicos de la educación temprana, en los tres contextos nacionales. Estas impresiones instantáneas experimentales identifican las prioridades del programa, las reacciones de los padres y niños, y el compromiso y preocupaciones de los maestros. Las conversaciones revelan similitudes culturalmente situadas de las prácticas en la educación temprana, pero también revelan diferencias entre contextos. Los maestros reportan sobre los desafíos de sus experiencias, pero también sobre los beneficios de su práctica y cómo se relacionan con los niños y sus familias. También se discuten ideas sobre la preparación para futuras pandemias.

## Introduction

In late December 2019 and early January 2020, a highly contagious, new virus engulfed residents of Wuhan, China. For many people, the virus initially seemed like a far-away menace of little consequence beyond the immediate area in which it emerged. However, amid warnings from global and national health officials and other scientists, it soon became apparent that this new virus, known as the novel coronavirus or COVID-19, was a worldwide threat to health, economic stability, and education, among many other economic consequences for individuals and their societies. This article represents a glimpse into national situations and the initial impact on preschools from the perspectives of early childhood teachers, administrators, and teacher educators at the time when all three countries had begun to relax their COVID-19 mitigation efforts.

As the virus hopscotched across the globe in the northern winter and spring of 2020, the authors communicated with early childhood colleagues in Sweden, Norway, and the United States to discuss the effects of the pandemic on preschools in their respective countries. Against the backdrop of frequent changes in infection and mortality rates, epidemiological understandings, suggested, or required mitigation strategies, including governmental policies on school closures, our informants for this report shared their own experiences, as well as additional experiences related to them by other early childhood professionals. These accounts provide snapshots of programmatic priorities and actions, parents’ and children’s reactions, and teachers’ commitments and concerns. These conversations revealed similarities and differences across contexts, teacher perceptions of the challenges and also benefits of various mitigation strategies, and teachers’ ideas about future preparedness.

Throughout this article, we use the term “preschool” to refer to all center-based early education and care provisions for children between birth and school-entry age. We also use the term “teacher” throughout this article to refer to caregivers, certified preschool teachers and other paid adults who supervise children in preschool settings.

## Overview of Responses to the Pandemic in Three Countries

During the first few months of the pandemic, responses to the virus in Sweden, Norway and the United States provide insights in the social, political, and educational landscapes in each country and the impact on parents, children, and early childhood educators. Both Sweden and Norway implemented national policies emphasizing the importance of working together for the common good (*dugnad* in Norwegian), but they did so in quite different ways. The US government produced voluntary guidelines but not national regulations, leading to piecemeal policies and practices across the country.

Sweden implemented the fewest changes, relying primarily on its citizens to follow government guidelines on social distancing and crowd size to avoid overwhelming the national health care system. People worked from home if possible. Visiting seniors in elder-care homes was prohibited. High schools and universities transitioned to on-line instruction; but preschools and primary schools remained open with few changes in routines, other than greater attention to cleanliness in school environments and increased emphasis on health-related practices like sneezing into one’s own sleeve and frequent handwashing. With few businesses closed and varying levels of compliance with social distancing practices, daily life in Sweden remains much the same as usual.

Norway’s policies were stricter than Sweden’s, with a nationwide “lockdown” closing preschools, schools and non-essential businesses. Norwegians worked from home whenever possible and were encouraged to walk or ride bicycles rather than using public transportation. To avoid overburdening medical services in less populated areas, citizens were prohibited from traveling away from their primary residences to vacation spots in the mountains or by the sea. Generally, citizens heeded King Harald V’s and Prime Minister Erna Solberg’s strong admonitions to take the situation seriously and to follow the lockdown orders. Daily life has changed considerably.

In the United States, national government officials produced a series of often-changing voluntary guidelines about social distancing, crowd size, and masks; but actual policy decisions were left largely up to state and local jurisdictions. In many places, daily life remains mostly unchanged; but in other places, social and work lives have changed quickly and drastically. The preschool described in this report is in California, where far-reaching restrictions initially included mandatory stay-home orders in some localities and closing all non-essential businesses and schools. Preschools are only allowed to serve children of essential workers, such as those in hospitals, the food supply chain, and public transportation if they can implement disease mitigation strategies, such as lower numbers of children per classroom, no interaction between children in different classroom cohorts, and prohibition of parents in the preschool sites.

In both Norway and Sweden, parents and children *have a right to early childhood education and care*, from infancy to school-entry age. Pre-primary education is not considered a right in the United States, where only a small percentage of preschools are fully funded by local, state, or national governments. This distinction is important, since the rights-based position of early education creates an imperative to keep preschools open to the greatest extent possible in Sweden and Norway. However, no such obligation exists in the United States, where discussions about school reopening often focus primarily on allowing parents to return to work to restart the ravaged economy and reducing the impact of the pandemic on the national elections scheduled for November 2020.

In all three countries, serious infection and mortality rates were highest among elderly and minority populations, as well as those with underlying conditions. Sweden and Norway have seen some job losses and economic impacts, but these outcomes are most dramatic in the United States. Previous research has documented the negative impact of extreme events—including epidemics, death, school closures, and loss of contact with primary caregivers—on the general wellbeing, mental and physical health, and educational outcomes of young children (e.g., de Walque 2011). Recent research indicates that the effects of school closures remain unclear and that other mitigation strategies may be more effective alone or in combination with school closings (Viner et al. 2020). Studies have also provided insights into children’s responses when schools reopen after epidemics (e.g., Rao 2006). Research has not yet been done on the non-medical effects of the current pandemic on young children. However, it is reasonable to assume that both the general impact of the pandemic on society and the particular impact of preschool closures will likely affect children, early childhood professionals, and families immediately and into the future.

In the following sections, a report from a preschool in Sweden focuses on the initial Crisis Action Plan, government limits on preschool guidelines, and staff efforts to balance their professional obligations with their concerns about their own health and the health of their families. The Norwegian report focuses on reopening strategies and staff development. The report from California focuses on establishing virtual classrooms and remote learning protocols to meet an increasingly high emphasis on academic outcomes for early education.

## Sweden: Report from a Head Teacher of a Parent-Cooperative Preschool

This report from Sweden is based primarily on information from the head teacher in a state-subsidized parent-cooperative preschool, where parents and teachers share responsibility for the overall organization and management of the school and where parents often work alongside staff to plan and implement daily activities and supervise children. When the seriousness of the pandemic became apparent, the preschool’s first priority was to work with the staff and the parent advisory board to develop a detailed Crisis Action Plan. It was assumed that people would pay closer attention to the guidelines in the early stages of the national response, so they wanted to act quickly. The goal was to create a resource to prevent the virus from entering the preschool and to be ready to reduce its spread if it managed to invade. The approach was to start with tight controls and then relax them as the situation permitted rather than going the other way around.

According to teachers, parents picture the preschool as a secure world, where children will always be safe no matter what, so staff realized they needed to address potential risks from the highly infectious virus right away. They also recognized that members of the school community, like society at large, held many different perspectives on the crisis and how it should be handled.

The plan calls for fundamental changes in level of parent participation, health and sanitation protocols, enrolment patterns, and teacher roles. Staff members revisit the plan every 2 weeks, making changes if new directives emerge from the Public Health Agency in Sweden (see: https://www.folkhalsomyndigheten.se/the-public-health-agency-of-sweden/) or if they discover better ways to make the preschool environment even safer.

This plan keeps daily preschool life as consistent as possible for children, except that teachers were supposed to build in time to talk with children about the pandemic, children must practice stricter hygiene, and their traditional end-of-year performance event will be conducted without an audience. However, the plan severely restricts parent presence in the preschool, creating an obvious contradiction with the core philosophy and practice of a parent cooperative.

When the school introduced the plan, they called upon parents to understand why these changes were necessary, to comply with the rules, and to anticipate modifications along the way. Initially, they expected parents on leave or working from home to keep their children at home with them. They also wanted children to stay home if their parents or siblings were ill. However, it turned out that preschools do not have the right to insist that children stay home in either of these circumstances. Directives from the National Agency of Education and Public Health Service emphasized that children should attend unless they are ill because preschool is such an important aspect of their early development and because preschool is a right for children and their families.

### Attendance and Staffing

During the first 2 weeks under the plan, many children stayed home. As time passed, attendance patterns returned to near-normal, even for children whose parents were at home.

Fortunately, the preschool staff remained in good health. However, it was apparent early on that several staff members and interns were experiencing stress and anxiety. Regular staff meetings provided an opportunity for them to talk openly about the risks of traveling to and from the center on trains and busses, as well as the possibility of exposure to the virus from close contact with children who might be carriers. They also worried about transmitting the virus back home to their own families. Voicing these concerns enabled the staff to encourage and support one another.

Some staff members felt that their right to protect themselves was, in essence, pitted against the children’s and parents’ right to preschool. However, they also understood that preschool is essential for children’s development and is especially important in times of crisis. Therefore, as a matter of professional ethics, the head teacher said, “Like other early childhood educators throughout the country, we do what we must do for the children and for our society.”

## Norway: Report from a Preschool

Preschools closed when the national shutdown began, in order to prevent the disease from spreading. Under agreements with the municipal emergency teams, only children of essential workers were allowed to attend. Maintaining communication with children and parents was a top priority during the closure, as was providing additional attention to children with special needs and families who expressed concerns about their child’s well-being. Many teachers transmitted videos from their preschools on private media channels they established. Some went the extra mile. For example, the day before Easter, teachers from one preschool went to children’s homes dressed in Easter Bunny costumes to wave to them through the windows. Preschool teachers and administrators met frequently, following social distancing guidelines, to support and encourage one another, participate in in-service training, and develop contingency plans for reopening. In describing their experiences, they frequently mentioned the importance of early education as a near-universal aspect of childhood and as a right that must be balanced against the need to protect early childhood professionals from a new and unrivaled enemy like COVID-19.

### Report from an Urban Preschool

Staff members continued to work during the shutdown. They took turns supervising a small number of children of essential workers on-site, trying to make the situation as normal as possible for them. At the same time, they needed to figure out how to use the digital staff handbook, digital deviation notifications, and various on-line platforms and applications. They created short videos of themselves singing and reading stories to the children. They also talked with all children and parents at least once during the 6-week shutdown and made more frequent calls to those who wanted more contact or needed it. They contacted the child welfare service and the health center about children and families who needed extra attention and support.

However, from the beginning they also focused on reopening. They needed to develop a common understanding of the health authorities’ requirements and how they would achieve them. They met with union representatives and the Parent Cooperation Committee. They completed an infection control course. They held reflection sessions to discuss their thoughts and feelings about the situation, how each of them was coping, and their strategies for meeting the children’s and families’ needs. They shared information about each child to make sure that they were reaching all of them through their various communication strategies.

They practiced various reopening scenarios. They discussed what they would do if a staff member or a child’s parent became infected; how they would help children follow the new hygiene and distancing protocols; what they would do if the children did not like the group to which they were assigned; how they would discuss the pandemic with the children, and how they would manage if a teacher became ill and unable to work. They made work-from-home and work-on-site schedules, delegating responsibilities among the staff.

### Reopening

After 6 weeks of lockdown, health authorities and political leaders announced that children and staff could safely return to preschool, primarily because COVID-19, at that point, was thought to affect children very rarely even though they were known carriers.

Initially, many parents expressed reservations about this decision. More than 15,000 people joined a Facebook group called, “My child should not be a test rabbit for COVID-19.” In response, the Ombudsperson for Children in Norway, Inga Bejer Engh, made this emphatic statement on prime-time television: “I would say to parents that it is now very important that you be guided by facts, not fear. And that you keep your head cool and listen to professional advice.” She recommended that families with persons who have chronic illnesses consult a physician before deciding whether to send children back to school. Evidently, her remarks were quite persuasive because most children returned as soon as preschools reopened.

The health and education authorities introduced safety guidelines for preschools, with the following three pillars as top priority:

1. Sick people (children, teachers, parents) should not be in preschool.
2. Always remember good hygiene.
3. Reduce contact frequency between people.

Preschools have had to implement several health protocols. Children were separated into small groups, called cohorts. The adult–child ratios were quite strict at first and then gradually became more flexible. Initially one adult was required for every three children under age three; but after the first 9 weeks, the ratio changed to 1:5. Children between 3 and 6 years old were initially in groups of six with one adult, but this ratio also became more flexible after several weeks.

The Norwegian Directorate for Education and Training established the following rules for staff when preschools reopened:

- Keep a distance of at least 1 meter from colleagues in other cohorts.
- Limit physical contact with children and staff during meetings and breaks.
- Use video conferencing instead of physical meetings with colleagues when possible.
- Avoid handshakes and hugging.
- Avoid wearing hand jewelry.
- After each use, thoroughly clean shared tablets, computers, and keyboards.
- Pay extra attention to hygiene around kitchens and eating spaces.
- Limit the use of public transport to and from the workplace when possible.
- Staff employed at multiple preschools must receive training and updates on protocols at each site. They should avoid working in more than one site on the same day if possible.

When time came to reopen, one teacher said, “I felt well prepared. The first day was a good day for the children and for me in spite of the changes.” The school day was shortened and all staffs were on hand so they could meet requirements for limited group size and lower adult–child ratios. All children arrived at the same time, which was new for them since arrival time was previously quite flexible. Their parents said good-bye to them at the gate rather than coming into the center as usual. Nevertheless, the children seemed happy to be back in preschool, adjusted quickly to their new groups, and eagerly *tried* to follow the new safety protocols, such as using hand sanitizers frequently and not darting into the space of another cohort to visit with a friend. To help with social distancing, teachers take their groups to a nearby park and to a nearby mountain area as often as possible.

While acknowledging the logistical challenges of the restrictions, teachers also commented on the benefits of smaller cohorts. They noted that they have longer and more frequent interactions with each child and that they are able to do even more than usual to follow-up on the children’s ideas and suggestions. For example, after a water play activity, the children wanted to learn more about different kinds of boats. A teacher said, “Because of the small-group situation, I was able to gather materials quite quickly so the children could construct their own boats and pursue their interest in watercraft, especially submarines.”

In spite of their overall positive feelings about working with smaller cohorts and distancing one cohort from another, some concerns have also arisen. For instance, teachers wonder how playing with only a few children each day will affect the children’s social development and coping skills. They wonder whether they made the best matches when they put the children into cohorts. They also admitted to some stress because they now have less time for planning and preparation. They also worry about logistics when one or more teachers must be absent. As a result, they will soon combine the cohorts into “departments” that include two or three groups. They will relax the boundaries between groups so that children can sometimes join friends in other cohorts, especially when children are outside.

Many parents have expressed their gratitude to the teachers, saying they feel confident that their children are as safe as possible when they are in preschool.

A teacher explained that, in many ways, the weeks since reopening have seemed extraordinary, “We keep our distance from colleagues, we wash our hands until they feel dry, we wave at the parents through the gate rather than talking with them, and we remind children of the new ‘rules’ they must follow. But in the most important ways, things have not really changed. We are a still team of teachers who cherish our time with children. We uphold our professional obligations to the children, their families, and the Norwegian society.”

## The United States: Report About Virtual Learning in Preschool and Kindergarten

Unlike Sweden and Norway, the United States has not implemented a cohesive national pandemic policy, leaving decision-making largely to each state. In California virtually all primary, secondary and tertiary schools closed when statewide stay-at-home orders went into effect and remain closed through the end of the academic year. Preschools are allowed to serve children of “essential workers” (defined in many different ways across the state), as long as they meet public health and licensing guidelines, such as strict disinfecting protocols; limiting classrooms to 10 children supervised by teachers who do not work with other cohorts; restricting children from coming into contact with peers from another classroom; social distancing by at least 1.83 m (6 feet). Most private non-profit, for-profit, and church-sponsored preschools that rely on tuition revenues have been forced to shut down because they are unable to meet the requirements and/or because parents are unable to continue paying tuition fees. Many of these schools now face the reality that reopening will be a struggle, if not an impossibility, if social distancing and limits on class size remain in effect much longer, leaving typically low-paid employees to seek government assistance if they can qualify for such assistance.

The Broadoaks School of Whittier College in Los Angeles is a private, non-profit laboratory/demonstration school with year-round programming for preschool through 8th grade. As a tuition-dependent enterprise, the school quickly realized they needed to do something extraordinary to maintain program quality and stabilize enrollment.

### Virtual Learning in Preschool

The preschool is now one of a few in the state (and perhaps, across the country) to offer 3 h of real-time, on-line programming per day for children between 2 and 5 years of age, in accordance with the children’s usual attendance patterns (2, 3 or 5 days per week). The consistent daily routine includes music and movement, story time, gross motor activities, free-choice time, and teacher-directed literacy, science and mathematics activities to address the preschool’s developmental and academic goals. Teachers email detailed information about virtual classroom activities and anticipated learning outcomes. Table 1 provides an example of what parents received for 1 day:

**Table 1:** Virtual classroom activities for preschool

*Zoom Lessons May 18, 2020*: high scope/key developmental indicator (KDI) D: language, literacy, and communication	
Activity/key developmental indicator	Description/material ideas^a^
*Monday* decorate your letter *Key developmental indicator* #27. Concepts about print: children demonstrate knowledge about environmental print *Vocabulary* initial, letter, outline *Questions* What is the first letter of your name? How will you decorate your initial? Could you decorate it in another way too?	*Description* children will decorate the first letter of their name using various art materials *Material ideas* bubble letter of their first initial, writing tools, glue sticks, and various art materials such as stickers, scraps of paper, pom poms, feathers, etc. *Challenge activity* decorate the rest of the letters in your name

^a^Material ideas are just suggestions. Feel free to use whatever you have handy. When in doubt ask your child. They have great suggestions!

Teachers have also created a private *You Tube* channel where they present language arts, STEM and social science activities for parents to use with their children to augment their experiences in the virtual classroom. Teachers recently added dramatic play to this resource. For the first session, the teacher asked the children to join her in pretending to be a doctor. She suggested that they find a doll or stuffed toy to be the patient and use toys or kitchen implements as pretend thermometers, stethoscopes, and other items that doctors need. Teachers plan to build on the children’s ideas and suggestions as they add more dramatic play activities to their *You Tube* video library.

### Remote Learning in Kindergarten

In California, children aged 5 and 6 years attend the first class in primary school, called kindergarten. During the pandemic closure, virtual kindergarten runs daily from 9:00 am to 2:30 pm, with breaks for play, snacks and lunch. Because such young children need adult support for on-line learning, parents receive detailed daily plans, downloadable materials, and links to a variety of additional age-appropriate resources for various content areas. The school archives live sessions so parents can access them if they are unable to attend when the virtual class is in session. Table 2 provides an example of the plan for 1 day, including links to learning activities the teachers created, as well as on-line materials from a variety of other resources. Descriptions of the material available through these links appear in italics.

**Table 2:** Information for parents on virtual classroom activities for kindergarten

*Kindergarten remote learning schedule* If you are not able to access a hard copy of an activity, your child can complete the work on paper or you can make a video of your child explaining the work and submit via email. Here is the link for those who cannot attend the real-time Zoom meeting: *Link* Google document with 24 slides detailing parent–child activities for 5 days. Additional links within slides provide information and materials about the literacy and math activities in the plan.	

*Plan for Monday* 5/18/2020	
9:00–9:45 9:45–10:15 10:15–11:00	*Morning message* (slide 2) *Links provided in the slide* (1) letter to parents explaining the importance of reading fluency, what reading fluency is, why it is important; (2) link to a reading passage (at the dentist) for the child to read with parent’s support; (3) additional information to parents on the importance of repetition and reading practice *Materials* at the dentist handout (optional) Nothing to submit to teachers
11:00–11:30	*Break—offline* practice your child’s sight words, math flashcards, or work on guided reading
11:30–12:15	*Reading group and materials* (slide 22) *Links provided in the slide* a commercial digital library used by the school and each class. Each child has a mailbox with assigned books for guided reading with the parent, as well as reading comprehension questions Nothing to submit to teachers
12:15–1:00 1:15–2:00 2:00–2:30	*Writer’s workshop* (slide 3) *Links provided in the slide* (1) explanation for parents on ideas and skills behind the ‘How-to write stories’ workshop; (2) video of teacher reading the storybook, ‘Everyone can learn to ride a bicycle’; (3) worksheet for a child to draw and/or write his/her own story; (4) word recognition sheet of important words associated with ‘how to’ stories (e.g., first, next, later, lastly) *Materials* explanation about writing ‘How-To’ stories; writing paper OR lined paper; List of transition words (optional); and a pencil

### First On-Line Meeting

The kindergarten teacher worked for days to prepare the first on-line session for 20 children, aged 5–6 years. Instructions had been sent home telling parents how to join the Zoom class at the appointed time. The teacher realized that virtual classroom would be a new experience for everyone and that the children would be extremely excited to see their friends, so she decided to begin with favorite songs and stories.

The moment came. The Zoom class opened. Immediately 11 children’s images popped up, indicating that their parents had successfully logged them in. The children began squealing and waving at one another, drowning out the teacher’s attempts to explain how to use the mute button and take turns speaking. Slowly, one-by-one, other children joined the session. As each new face appeared on the screen, the children again began squealing and shouting “hello.” By the time the teacher electronically “closed the door” to the virtual classroom, a full 15 min had passed.

By now some children were jumping on the sofa, rolling around on the floor, or bringing their pets to class, often disregarding their parents’ instructions to calm down and listen to the teacher. One child’s sibling strolled in front of the camera wearing only underpants, igniting laughter from the children—at least the ones who were still paying attention. When the session ended, the teacher felt as if she’d been teaching for hours rather than 45 min. She was amused by the children’s unbridled excitement over seeing one another again. She empathized with the parents’ frustrations with the technology and their children’s behavior. She recognized that she needed to control the microphones herself in future meetings. She said, “It was utter chaos but I was so happy to see the children again.”

Things quickly got better as the teacher and the children adjusted to remote learning. The teacher complies with the school-wide requirement to record all on-line sessions for review by school administrators and to ensure the children’s safety during virtual school. She and the principal regularly discuss the challenges of completing the skill-specific, year-end report card when there is no way to fairly assess each child’s progress and performance without in-person contact.

Like their counterparts across the state, Broadoaks teachers consistently report that remote teaching seems more challenging and requires much more advanced planning than teaching during “normal” times.

With so much ambiguity about the future, teachers are already developing several contingency plans for the next school year, including on-site teaching, remote teaching, or a hybrid model.

To express their appreciation, parents recently drove their children around the school parking lot, honking their horns and displaying thank you signs for teachers and staff. Echoing the sentiments of her colleagues, one teacher said, “This just filled my heart. I can’t wait until we can all be together in the classroom again, even if we have to wear masks, smell like disinfectant, and keep six feet apart.”

### Access, Privacy, and Safety Concerns

Teachers and administrators in public and private settings across California face several hurdles during school closures. For example, they must figure out how to provide equitable educational experiences for children who do not have computers or access to the Internet. Consistency of attendance and home support for on-line learning have been frequent challenges, especially for children whose parents are working at home or struggling with financial problems, children from two-home families due to divorce or separation, and families with children with special needs.

A variety of privacy and on-line safety issues have come up as well. Some parents are not comfortable with images of their homes and family members being visible during on-line instruction. Additionally, parents worry that children may inadvertently encounter inappropriate material or come into contact with predators on-line. New ideas to address privacy concerns appear frequently on Internet sites for teachers and parents. For example, some teachers asked families to create pseudonyms to protect children’s identities on-line. Parents in one class suggested some amusing names, such as Pandemica (*pandemic*), Cloroxia (*spoof of brand name of popular bleach*), Vyrus (*spelling variation for virus*), Korona (*close to corona, as in coronavirus*), Quarantina (*quarantine*), Charmin (a toilet paper brand name), and the twins, Sochal and Distauce (*for social distance*). Additional examples appear on You Tube.

## Discussion

Despite differences in official responses to COVID-19 in Sweden, Norway, and the United States, specifically regarding preschool closures, several similar themes emerged in teachers’ descriptions of the pandemic’s impact on early education in their countries. In addition, preschools in all three countries were using similar virus mitigation measures, such as extra handwashing, smaller group sizes, lower teacher–child ratios, stricter sanitation requirements, prohibiting parents from entering the preschools, and social distancing, though there are some differences in distancing requirements (e.g., at least 1 m in Norway and at least 1.83 m in the United States). Informants in Sweden and Norway did not mention the use of masks in preschool, a controversial practice in some previous health crises, especially in Asia (Rao 2006). Use of masks by teachers and/or children remains an open question in California as reopening guidelines are being developed.

Informants in all three countries emphasized the benefits of preschool for children’s overall wellbeing, social development and early learning, as well as its important role in providing childcare for working parents. They expressed concerns about the possible effects upon children of extended school closures, as well as social distancing, limited group size, separation of cohorts, and other disease mitigation methods. They expressed particular concern about children with special needs and those who are especially vulnerable due to other life circumstances.

Teachers in Norway and Sweden described ethical struggles arising from their desire to safeguard their own health while also living up to their professional responsibilities to children and families. Teachers in Norway and California commented about the loss of in-person contact with parents and children when preschools were closed, explaining that they view these relationships as the heart and soul of teaching. Informants in California emphasized concern about the children’s falling behind academically, especially given increasingly high outcome standards for preschool and early primary grades. Broadoaks teachers also recognized that their virtual preschool and kindergarten classrooms relied heavily of parents’ having enough time to implement their on-line lessons. Teachers in other tuition-reliant preschools in the United States have additional worries about the survival of their schools, as well as the possibility of pay cuts and job losses.

Consistent with an earlier study on pandemic preparedness in child care centers (Shope et al. 2017), informants from all three countries felt ill-prepared for the changes required of them when the pandemic struck, ranging from the logistics of implementing new hygiene and sanitation protocols to the technological demands of creating videos, establishing media channels, and setting up virtual classrooms. They also expressed uncertainty about how they could sustain protocols for frequent sanitizing of classroom furnishings and materials while also meeting their other responsibilities and how they could enforce social distancing requirements without constantly nagging the children.

None of the informants seemed familiar with guidelines published after previous epidemics and pandemics (e.g., Centers for Disease Control and Prevention 2006, 2014; Santa Clara County Office of Education and Public Health Department n.d.). Teachers in California mentioned that they routinely have in-service training and practice drills for fires, earthquakes, and active shooters. However, their experience with disease control has focused on routine hygiene and notifications to parents when a child or staff member contracts a contagious illness.

Teacher responses also illustrated the significance of the broader cultural landscapes in which preschools tackled the pandemic. For instance, in Sweden and Norway, governmental policies and informant responses reflected their commitment to early education as a right for children and families, a position that early childhood professionals, parents, and the general public in the Nordic countries have strongly endorsed for several decades (see Wagner et al. 2020). However, when faced with the possibility of exposure to the virus from child carriers, staff members had varying reactions to governmental directives about keeping schools open in Sweden and reopening them in Norway. In some ways, these directives pitted children’s and families’ right to preschool against the teachers’ right to protect themselves. As one teacher explained, where one stands in this particular debate, depends on how one balances the known benefits of early education against the known and unknown health risks to preschool personnel.

Teachers in the two Nordic countries emphasized preschool as a right with fundamental value to society as a whole, while informants in California focused primarily early education’s academic value to children and its role in allowing the economy to recover. On-line privacy and security was an important issue in the United States, but was not mentioned as a concern by Nordic informants.

## Implications

Teachers in all three countries pointed to “silver linings” amidst the pressures of dealing with the pandemic. For example, teachers in Norway noted that, as a result of smaller group sizes and more favorable adult–child ratios, they are able to interact with each child more frequently and to follow-up on children’s interests more fully. They said they plan to incorporate similar opportunities into preschool life after the restrictions are lifted. Teachers who experienced school closures in Norway and the United States noted that circumstances forced them to develop new technology skills that will be useful when things return to normal.

Teachers also commented on the need for more thorough advanced planning in preparation for future crisis situations. Teachers also suggested that, after the current crisis ends, meetings should occasionally be held virtually so teachers and parents remember how to use the technology and become familiar with updates. Program administrators and teacher educators thought it could be helpful for staff to familiarize themselves with new pandemic guidelines emerging in the wake of COVID-19 (e.g., Centers for Disease Control and Prevention 2020; Los Angeles County Department of Public Health 2020; Public Health Agency of Sweden 2020), including those addressing specific issues, such as protocols for school openings (Education International 2020) and protecting children from violence during crises (End Violence against Children 2020), since family stress and economic vulnerability increase the risk of violence in homes and decrease children’s opportunities to seek help from teachers, social workers, and other support systems.

## Conclusions

In the authors’ opinion, the reports from Sweden and Norway are reasonably representative of common preschool experiences and practices during the first few months of the pandemic, while the report from the United States focuses on one unique example.

Time and future research will shed light on the benefits and risks of preschool closures and mitigation practices, as well as on the impact of societal stressors upon children. However, the dedication and professionalism revealed by the early childhood educators who participated in these discussions suggested that preschool teachers will be capable of meeting the various challenges that may come their way.
